# High prevalence of enterotoxigenic *Escherichia coli* strains in hospitalized diarrhea patients: a preliminary study from a cholera-endemic area in India (2022)

**DOI:** 10.3389/fmicb.2025.1470783

**Published:** 2025-06-04

**Authors:** Hemant Kumar Khuntia, Prabir Manna, Deepak Kumar Barik, Subhojeet Biswas, Prasanta Kumar Bramha, Sanghamitra Pati, Manoranjan Ranjit, Madhusmita Bal, Anna Salomi Kerketta

**Affiliations:** ^1^Institute of Veterinary Science and Animal Husbandry, Department of Microbiology, Siksha ‘O’ Anusandhan University, Bhubaneswar, Odisha, India; ^2^Indian Council of Medical Research (ICMR)-Regional Medical Research Centre, Bhubaneswar, Odisha, India

**Keywords:** diarrhea, *E. coli*, ETEC, *est*, *elt*, cholera, *V. cholerae*

## Abstract

**Introduction:**

Enterotoxigenic *Escherichia coli* (ETEC), producing heat-stable (ST) and/or heat-labile (LT) enterotoxins, is a major cause of diarrhea in children and travelers in developing countries. Surveillance in cholera-endemic regions is crucial for timely public health response.

**Methods:**

Between May and November 2022, a cross-sectional study was conducted in Puri, India. Rectal swabs from 256 hospitalized diarrhea patients were analyzed for diarrheagenic *E. coli* and *Vibrio cholerae* using microbiological and molecular methods. Antimicrobial susceptibility of ETEC isolates was also assessed.

**Results:**

ETEC was detected in 20.3% of cases, making it the most prevalent pathogen. No Vibrio cholerae was isolated. EAEC and EPEC were identified in 6.2% and 2.3% of cases, respectively. ETEC was significantly more common in children under 2 years (*p* < 0.00001), though overall age correlation was weak (*R* = –0.013). Symptoms included watery stool, vomiting, abdominal pain, and dehydration, with dehydration significantly associated with adults (*p* < 0.05). ETEC strains were susceptible to streptomycin, chloramphenicol, gentamicin, and amikacin.

**Discussion:**

ETEC has emerged as the dominant diarrheal pathogen in a cholera-endemic area, posing a risk to both children and adults. These findings highlight the need for continued epidemiological monitoring and age-targeted interventions.

## 1 Introduction

Diarrhea remains a significant global health challenge, particularly in developing countries, where it contributes to high morbidity and substantial mortality. It is caused by a wide range of enteropathogens, including bacteria, viruses, and parasites. Among different bacterial pathogens, diarrheagenic *Escherichia coli* (DEC) plays a key role in disease occurrence (Mokracka et al., 2011; Lozer et al., 2013).

DEC is categorized into six pathotypes: namely enterotoxigenic *E. coli* (ETEC), enteroaggregative *E. coli* (EAEC), enteroinvasive *E. coli* (EIEC), enteropathogenic *E. coli* (EPEC), enterohemorrhagic *E. coli* (EHEC), and diffusely adherent *E. coli* (Mokracka et al., 2011; Lozer et al., 2013). Among these pathotypes, ETEC is a major causative agent of diarrhea in children under 5 years of age in developing countries (World Health Organization, 1999; Rao et al., 2003). In addition, it is a well-known cause of traveler’s diarrhea, affecting visitors to endemic regions such as sub-Saharan Africa (West and East Africa), North Africa, and Western Asia (Lopez-Velez et al., 2022; Steffen et al., 2015).

ETEC is responsible for an estimated 220 million diarrheal episodes annually, including approximately 75 million cases in children under 5 years of age (Anderson et al., 2019), with mortality in this age group ranging between 18,700 and 42,000 deaths per year (Khalil et al., 2021; Anderson et al., 2019; Begum et al., 2014; Bourgeois et al., 2016). In endemic regions, ETEC was responsible for approximately 100 million diarrheal cases and 60,000 child deaths in 2015 (Bagamian et al., 2020).

ETEC transmission occurs primarily through the ingestion of contaminated food and water, with transmission exacerbated by poor sanitation, unsafe drinking water, and open defecation, with conditions that are prevalent in many low-income countries.

Diarrhea caused by ETEC infection results from the production of heat-labile enterotoxins (LT) and/or heat-stable enterotoxins (ST), which are encoded by *elt* and *est* genes, respectively. Some strains (*elt*/*est*) of ETEC produce both the toxins simultaneously (Finkelstein et al., 1976). The clinical manifestation of ETEC diarrhea is characterized by the sudden onset of cholera-like watery diarrhea, which can often lead to rapid dehydration (Finkelstein et al., 1976). Additional symptoms may include vomiting, stomach cramps, fever, and headache (Youmans et al., 2015). Seasonal peaks in ETEC prevalence varies by geographical regions (Levine et al., 2012; Paredes-Paredes et al., 2011).

While the global burden of ETEC highlights its importance as a public health priority, translating this knowledge into localized surveillance and intervention is critical to mitigate its impact. Puri is a coastal city in Odisha, India, and a major religious tourism hub. It experiences recurrent diarrheal outbreaks each year, affecting both residents and visitors. Factors such as brackish water reservoirs, substandard water quality, inadequate sanitation, and high population density contribute to the endemic nature of diarrheal diseases in this region. Historically, *Vibrio cholerae* (*V. cholerae*) has been recognized as the primary etiological agent of these outbreaks (Samal et al., 2008; Kerketta et al., 2019); however, the contribution of ETEC has remained poorly explored. Given emerging evidence of a shifting etiological landscape, investigating the local epidemiology of ETEC in endemic settings such as Puri is essential to implement region-specific public health strategies that align with global priorities.

This study aimed to investigate the prevalence of ETEC in Puri in 2022 and examine its toxin profile, antibiotic resistance patterns, distribution across different age groups, and seasonal trends. The findings will contribute to a better understanding of the epidemiology of ETEC in Puri, which will inform public health interventions to mitigate its impact.

## 2 Materials and methods

### 2.1 Study design

This study is part of an ongoing hospital-based diarrhea surveillance program across three health facilities in Puri that is aimed at detecting diarrhea-causing etiological agents in hospitalized patients with acute diarrhea. This study particularly focused on the detection of ETEC and their antibiogram.

### 2.2 Study setting and population

The present study has been ongoing since 2011, following the administration of Shanchol—a bivalent oral cholera vaccine (OCV)—to a cholera-vulnerable population in the Satyabadi Block of Puri district. The objective was to evaluate the vaccine’s efficacy by administering two doses through the existing public health infrastructure, following the first round of clinical trials conducted in Kolkata (Kar et al., 2014). This surveillance study was conducted in the following three hospitals in 2022: Infectious Disease Hospital (IDH), Puri; Pediatric Hospital, Puri; and Area Hospital in Satyabadi Block, Puri. Due to its religious significance, Puri receives thousands of pilgrims every day from across the country and around the world. The resulting overcrowding creates unhygienic conditions, leading to the emergence of diarrhea, particularly during the rainy season. Puri is surrounded by numerous rural villages to the northeast and northwest, with a catchment population of more than 150,000. Both local inhabitants and tourists in Puri constitute the vulnerable population at risk for diarrhea.

### 2.3 Clinical specimen

In Odisha, each year, higher rates of diarrhea are expected from the month of June to the month of November, corresponding with the pre-monsoon, monsoon, and immediate post-monsoon periods. These months are marked by elevated temperatures, heavy rainfall, and flooding, which contribute to unsafe water and poor sanitation, thereby increasing the risk of diarrheal diseases. Considering this period as the high-diarrhea season, our study was designed to collect rectal swab samples once a week from the three health facilities in Puri District.

According to the WHO guidelines, diarrhea was defined as the passage of three or more loose or liquid stools within any 24-h period during the 3 days before presentation or the passage of one or two loose/liquid stools accompanied by any signs of dehydration (World Health Organization, 2005). Patients with chronic gastrointestinal disorders (e.g., Crohn’s disease, ulcerative colitis) that cause recurrent diarrhea and those who had recently used antibiotics were excluded. From May to November 2022, we enrolled hospitalized patients who presented with acute diarrhea. During the investigation, non-randomly selected rectal swab samples were collected from the hospitalized patients with acute watery diarrhea, prior to the administration of antibiotics, and stored in Cary–Blair transport (CBT) medium. Due to administrative constraints and limited manpower, samples were collected once a week during routine hospital visits by staff from the Regional Medical Research Centre (RMRC) who traveled from Bhubaneswar (approximately 60 km away). As a result, non-random sampling was adopted to maximize patient enrolment during these scheduled visits, especially in the challenging post-COVID period. Demographic data were collected using pre-structured forms, with the consent of each patient or attendant. The patient’s dehydration status—categorized as none, mild, moderate, or severe—was examined and assessed by a physician based on clinical signs, as described by the WHO. A pre-structured questionnaire covering demographic background, medical history, and previous treatment was completed by the patient or their attendant.

### 2.4 Bacteriological analysis of the rectal swab samples

Immediately after collection, the rectal swab samples in CBT were transported to the Indian Council of Medical Research (ICMR)-RMRC Microbiology Laboratory for processing. The rectal swabs were inoculated onto thiosulphate–citrate–bile salts–sucrose (TCBS) agar, MacConkey agar, and Hektoen enteric agar, followed by enrichment in Alkaline Peptone Water (APW) to isolate *V. cholerae* and other major diarrhea-causing pathogens. Typical colonies were selected and tested biochemically to identify major diarrhea-causing bacterial pathogens using conventional methods.

### 2.5 PCR detection of virulent genes in DEC

At least three typical pink colonies on MacConkey agar were selected per sample for DNA extraction using the boiling method, as described in a previous study (Dutta et al., 2013). *E. coli* strains were confirmed genetically by detecting the *alr* marker gene using a monoplex PCR assay (Yokoigawa et al., 1999). The *E. coli* strains that tested positive for the *alr* gene were subjected to a multiplex PCR assay for the detection of various virulent genes for the identification of DEC. The multiplex PCR assay was conducted to detect DEC pathotypes: ETEC (*elt* and *est*), EPEC (*eae* and *bfpA*), and EAEC (*aatA*), using specific primer pairs described previously (Panchalingam et al., 2012). PCR amplification was performed using the appropriate volumes of 10x amplification buffer (500 mM KCL, 100 mM Tris HCL, 15 mM MgCl2, PH-8.3), 2.5 mM of each deoxynucleoside triphosphate, 10Pmole of each primer, 1.25 units of Taq DNA polymerase (Mumbai, India: HiMedia Laboratories, LLC), and 5 μL of template DNA. The reaction volume was adjusted to 25 μL using sterile triple-distilled water. The PCR assay was performed in an automated thermal cycler (Mumbai, India: HiMedia Laboratories, LLC) for 30 cycles using conditions described previously (Dutta et al., 2013). Aliquots of the PCR product were analyzed using agarose gel electrophoresis (1.8% wt/vol) in Tris/Borate/EDTA buffer, stained with ethidium bromide, and visualized under UV illumination. Positive controls for ETEC, EPEC, and EHEC and a negative control, *Salmonella enterica serovar Typhi* (Provided by Dr. Asish Kumar Mukhopadhyay, the National Institute of Cholera and Enteric Diseases (NICED), Kolkata), were included in each PCR assay set. The PCR method was initially standardized at the NICED, Kolkata, using specific primers targeting the virulence genes of toxigenic *E. coli* strains.

### 2.6 Antibiotic susceptibility

Antimicrobial susceptibility testing of the isolated DEC was performed on Mueller–Hinton agar using the Kirby–Bauer disc diffusion method [the Clinical and Laboratory Standards Institute (CLSI), Wayne, PA, 2006] with a panel of antibiotic discs: tetracycline (T 30 μg), gentamicin (GM,10 μg), streptomycin (S 10 μg), chloramphenicol (C, 30 μg), amikacin (AK, 30 μg), norfloxacin (NX 10 μg), neomycin (N, 30 UI), ampicillin (AM, 10 μg), ciprofloxacin (CP, 5 μg), ofloxacin (OF, 5 μg), furazolidone (FZ, 100 μg), azithromycin (ATZ, 15 μg), and doxycycline (DO 30 μg). According to the Clinical and Laboratory Standards Institute (CLSI) guidelines (Albert et al., 1995), susceptibility was categorized as sensitive or resistant based on the diameter of the zone of inhibition. Toxigenic *E. coli* positive control strains were provided as a gift by Dr. Ashish Mukhopadhya from the National Institute of Cholera and Enteric Diseases (NICED), Kolkata.

### 2.7 Statistical analysis

Data analysis was conducted using the software SPSS (version 27), R (version 4.3.1), and Python (version 3.10). Descriptive statistics, including means, standard deviations, and frequencies, were used to summarize the data. For inferential analysis, specific tests such as independent *t*-tests, a one-way ANOVA, and the chi-squared tests were employed to assess differences between the groups. The assumptions of normality and homogeneity of variance were evaluated using the Shapiro–Wilk test and Levene’s test, respectively. When these assumptions were violated, non-parametric alternatives, such as the Mann–Whitney U test and the Kruskal–Wallis test, were applied. Correlations were assessed using either Pearson’s or Spearman’s correlation, depending on the data distribution. A regression analysis was also performed, where appropriate, to examine predictive relationships. Statistical significance was set at a *p*-value of < 0.05. The correlation between ETEC prevalence and age was assessed using Pearson’s correlation to determine the strength and direction of the relationship. Pearson’s correlation was implemented as it measures the linear association between two continuous variables. The analysis was performed using SPSS, R, and/or Python, and statistical significance was set at a *p*-value of < 0.05. The correlation coefficient (*R*-value) and *p*-value were calculated to interpret the relationship.

### 2.8 Ethics statement

The ethical committee of RMRC, Bhubaneswar approved the study. Written informed consent was obtained from each patient before the collection of samples. Guardians provided written consent in cases of children.

## 3 Results

During the study period from May to November 2022, a total of 256 rectal swab samples were collected from acute diarrhea patients admitted to three hospitals in the Puri district of Odisha. All of these patients met the inclusion criteria and were enrolled in the surveillance study. The age of the hospitalized patients ranged from 0 to 80 years. A total of 100 (39%) patients were aged <5 years, and 156 (61%) patients were aged >5 years. Among the enrolled patients, 45.4% were male patients and 54.6% were female patients, with a median age of 30 years. This distribution reflects the greater vulnerability of these age groups to diarrheal diseases. A significant proportion of participants (84.40%) were residents of rural areas surrounding the hospitals, while the remaining (15.60%) were from urban areas within Puri town.

### 3.1 Incidence of DEC in the studied patients

**Figure 1:**
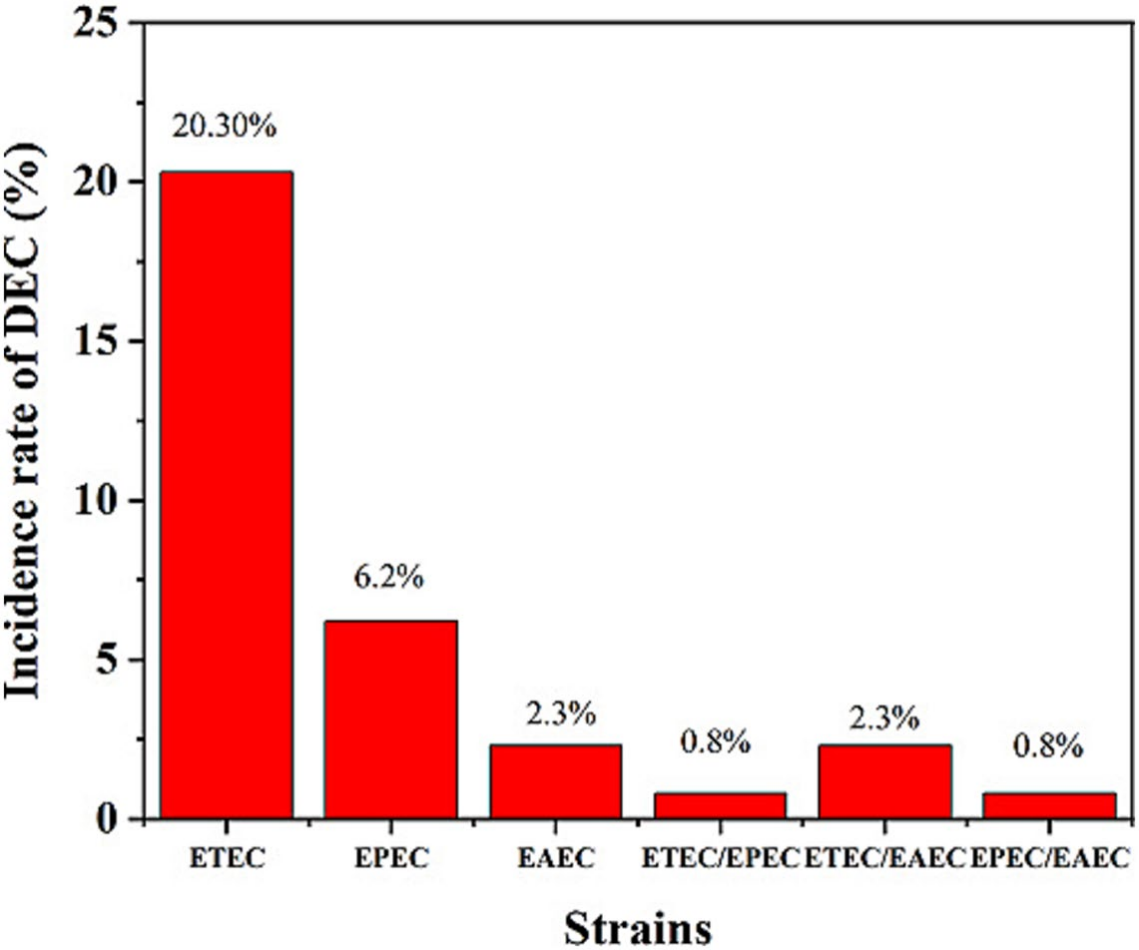
Incidence rates of DEC strains among the patients. ETEC was the most prevalent strain (20.3%), followed by EPEC (6.2%) and EAEC (2.3%). Mixed infections (ETEC/EPEC, ETEC/EAEC, and EPEC/EAEC) were also detected at lower rates.

A bacteriological analysis of the 256 rectal swabs revealed the following pathogens: *E. coli* spp. (172, 67.2%), *Shigella* spp. (12, 4.7%), *Salmonella* spp. (four, 1.7%) but no *V. cholerae* isolates. Of the total 172 presumptive *E coli* spp., 108 (62.8%) were genetically confirmed as *E. coli* through the detection of the *alr* gene. Of the 108 genetically confirmed *E. coli* spp., 72 (66.7%) were identified as DEC. Overall, 29% (72/256) of the total patients were DEC-positive, confirmed by the detection of different virulent genes in *E. coli* strains. Among the total samples, ETEC strains (20.3%, 52/256) were identified as the predominant pathogen, followed by EAEC (6.3%, 16/256) and EPEC (2.3%, 6/256) (Figure 1). Of the total DEC-positive cases, ETEC accounted for the highest proportion, 70.3% (52/72), as the predominant diarrhea-causing pathogen. Genetic screening did not identify any strains belonging to EHEC. Combinations of infections were detected among patients with diarrhea, with the following combinations: ETEC/EPEC 0.8% (2/256), ETEC/EAEC 2.3% (6/256), and EPEC/EAEC 0.8% (2/256) (Figure 1).

### 3.2 Statistical analysis

The statistical analysis revealed a significant association between the specific DEC strains and the age groups of patients with diarrhea (Table 1). ETEC was significantly more prevalent among children aged 0–23 months compared to those aged ≥15 years (chi-squared = 36.70, *p* < 0.00001). The odds of ETEC infection among children aged 0–23 months were 8.56 times higher than those in the ≥15-year group (95% CI: 2.77–26.43). Both EPEC and EAEC exhibited significant age-related differences (chi-squared = 14.19, *p* = 0.003 for EPEC; chi-squared = 19.14, *p* = 0.00026 for EAEC). However, these strains were less prevalent in the younger age groups compared to the older patients, with odds ratios of 0.07 (95% CI: 0.004–1.28) and 0.24 (95% CI: 0.07–0.86) for EPEC and EAEC, respectively. Mixed infections (e.g., ETEC/EPEC and EPEC/EAEC) did not demonstrate significant associations with the age groups, potentially due to smaller sample sizes. A negligible negative correlation was observed between age and ETEC prevalence (*R* = −0.013), indicating that ETEC occurrence did not substantially vary across the age groups. This finding suggests that ETEC prevalence does not significantly change with increasing age.

**Table 1:** Statistical summary of age-associated prevalence of DEC.

Strain	No. of age groups compared	Age groups compared	Chi-squared	*P*-value	Odds ratio -23 m vs. ≥15 yr	95% CI	Interpretation
ETEC	4	A	36.70	**<0.00001**	8.56	(2.77–26.43)	Significantly more prevalent in infants
EPEC	4	A	14.19	**0.003**	0.07	(0.004–1.28)	Less prevalent in infants; significant age association
EAEC	4	A	19.14	**0.00026**	0.24	(0.07–0.86)	Less prevalent in infants; significant age association
ETEC/EPEC	4	A	4.55	0.21	0.24	(0.01–5.42)	No significant association; possibly due to the small sample size
ETEC/EAEC	4	A	3.96	0.27	4.25	(0.18–97.83)	No significant association; wide CI suggests limited data
EPEC/EAEC	4	A	4.55	0.21	0.24	(0.01–5.42)	No significant association; possibly due to the small sample size

*p*-values in bold font represent statistical significance. This table summarizes the statistical association between the age groups and the prevalence of diarrhea genic *E. coli* (DEC) strains. The results are based on chi-squared tests. Odds ratios (OR) compare the prevalence in infants (0–23 months) to adults (≥15 years). Bold *p*-values indicate statistically significant associations (*p* < 0.05). Age Group A includes 0–23 months, 24–59 months, 5–14 years, and ≥15 years. OR, Odds Ratio (comparing 0–23 months to ≥15 years); CI, Confidence Interval.

### 3.3 Virulent gene distribution in the DEC pathotypes

Among the different types of DEC, the potential pathotype was determined based on the higher prevalence of virulent genes, either individually or in combination with other genes (Table 2). In the present study, the prevalence of *est* was 14.8% (38/256), followed by *elt* at 11.7% (30/256) and a mix of *elt/est* at 6.2% (16/256). Overall, ETEC (20.3%, 52/256), harboring *elt, est,* and *elt/est,* was identified as the dominant pathotype strain-causing diarrhea. In the case of EPEC, only 2.3% of the samples (6/256) amplified *eae,* and no *bfpA,* either alone or in combination with *eae,* was detected. This finding indicates that the patients in our study were infected with atypical EPEC strains, while no typical EPEC strains were prevalent. Among the EAEC isolates (*n* = 16), all harbored the *aatA* gene, representing 6.25% (16/256) of total samples that tested positive for *aatA*.

**Table 2:** Genotypes and distribution of various virulent genes of DEC among the different age groups in the study population (*n* = 256).

Strain type	0–23 mo	24–59 mo	5–14	≥15	Total
ETEC	8	16	–	28	52
*elt*	4	4	–	22	30
*est*	4	16	–	18	38
*elt+est*	–	4	–	12	16
EPEC				6	6
*eae*	–	–	–	6	6
EAEC, *aatA*	4	–	–	12 12	16 16
ETEC/EPEC *elt+est+eae*	–	–	–	2 2	2 2
ETEC/EAEC *elt+aatA* *elt+est+aatA*	2 2	–	–	4 2 2	6 4 2
EPEC/EAEC *eae+aatA*	–	–	–	2 2	2 2

This table shows the presence of key virulence genes in DEC pathotypes—ETEC (*elt*, *est*, *elt+est*), EPEC (*eae*), EAEC (*aatA*), and their combinations—across the four age groups. Values indicate the number of positive cases per gene or gene combination. The total count for *elt, est, eae,* and *aatA* includes both single and mixed gene amplifications.

### 3.4 Age- and sex-wise distribution of ETEC

Among the different DEC pathotypes, ETEC showed the highest prevalence at 20.3% (52/256) in our study. Age-specific distribution revealed that ETEC was detected in children under 5 years and adults over 15 years, with no isolates of ETEC observed in the 5–15-year age group—possibly due to the smaller number of samples or natural immunity within that age range (Table 2). Children under 5 years accounted for 9.4% (24/256) of the ETEC cases compared to 11% (28/256) in the adults. The difference was minimal and not statistically significant. Overall, the prevalence of ST-ETEC (14.8%, 38/256) was higher compared to LT-ETEC (11.8%, 30/256) across all age groups. Age-specific distribution of ST- and LT-ETEC showed that LT-ETEC was more prevalent in adults (8.6%, 22/256) compared to children under 5 years (3%, 8/256). ST-ETEC was detected in 7.8% (20/256) of children and 7% (18/256) of adults, indicating a negligible difference. Among adults, LT-ETEC (8.6%) was slightly more common than ST-ETEC (7%), although the difference was small. In contrast, among children under 5 years, ST-ETEC (7.8%, 20/256) was more prevalent than LT-ETEC (3%, 8/256). A higher prevalence of ETEC infection was observed in male participants (75%) compared to female participants (25%). Of the total ETEC enterotoxin-positive cases, ST accounted for 45.3%, LT for 35.7%, and the combination of ST/LT for 19% (Table 2). A higher proportion (58.8%, 40/68) of single enterotoxin-producing strains—either ST or LT—was observed in adults (>15 years). Among adults, the prevalence of LT (42.4%) was higher than that of ST (34.6%); however, among children under 5 years, the prevalence of ST (29.4%) was higher than that of LT (11.7%) (Table 3).

**Table 3:** Toxin profiles of ETEC isolated from the diarrhea patients across different age groups.

Toxin type	Age of the participants	No (%)			
	0–23 mo	24–59 mo	5–14 yr	≥15 yr	Total
ST	4(50)	16 (66.7)	–	18 (34.6)	38 (45.3)
LT	4(50)	4 (16.7)	–	22 (42.3)	30 (35.7)
LT/ST	–	4 (16.7)	–	12(23)	16 (19)
Total	8 (100)	24 (100)	–	52 (100)	84 (100)

The toxins found in our ETEC isolates are ST, LT, and ST + LT. This table presents the number and percentage of ETEC isolates producing heat-stable (ST), heat-labile (LT), or both toxins (ST + LT) across the four age groups. No ETEC isolates were detected in the 5–14-year age group. Percentages represent the proportion of each toxin type within each age group.

### 3.5 Clinical symptoms of the ETEC pathotype in adults and children

The observation of clinical symptoms in the ETEC-infected children and adults with acute diarrhea revealed that watery stool was the most common symptom among almost all children and adults (Table 4). It was observed that a higher percentage of children experienced vomiting (81%) and fever (14%) compared to the adults, who exhibited vomiting in approximately 75% of cases and fever in 6%. Upon admission, a higher proportion of ETEC-infected adults exhibited severe (36%) and moderate (45%) levels of dehydration compared to the pediatric patients. Intravenous (IV) rehydration therapy was required in 54% of the adults compared to 13% of the children, while the remaining cases in both groups were managed with oral rehydration. Approximately 50% of the ETEC-infected patients experienced abdominal pain.

**Table 4:** Clinical features, dehydration, rehydration mode, and toxin profiles of ETEC-positive patients by age group (*n* = 52).

Age group	No of ETEC cases	Vomiting (%)	Fever (%)	Abdominal pain (%)	Watery stool (%)	Non/Mild dehydration	Moderate dehydrati-on (%)	Severe dehydration (%)	Oral rehydration (%)	IV rehydration (%)	ST ETEC	LT-ETEC	ST/LT
0–23 months	08	81%	14	50	88	88	12	0	87	13	4	4	0
24–59 months	16	00	00	00	94	81	19	0	88	13	16	4	4
5–14yr	–	–	-	–									
≥15 years	28	75	6	50	96	28	45	36	46	54	18	22	12
Total number, *n* = 52	52	–	–	–	95	54	33	19	65	37	38	30	16

### 3.6 Seasonality of ETEC

**Figure 2:**
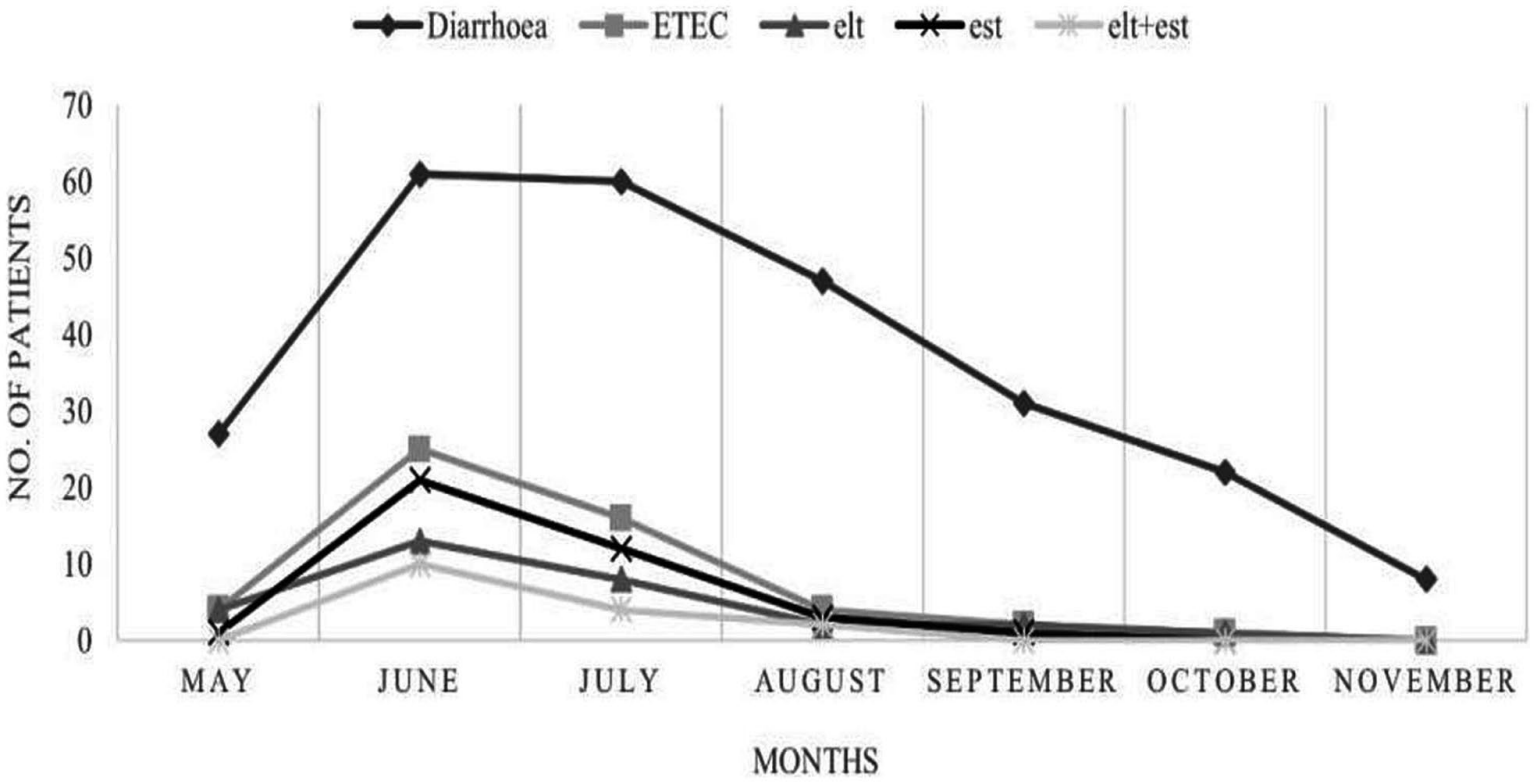
Seasonal incidence of ETEC harboring virulent genes *elt, est,* and *elt+est.* Diarrheal cases and ETEC detection peaked in June and July, with a decline observed from August to November. The toxin genes (*elt*, *est*, and *elt+est*) followed a similar seasonal pattern.

Diarrhea caused by ETEC was observed from May to October 2022. The month-wise distribution of ETEC strains in the samples was as follows: 14.8% (4/27) in May, 44.3% (27/61) in June, 26.7% (16/60) in July, 4.2% (2/47) in August, 6.4% (2/31) in September, 4.5% (1/22) in October, and 0% (0/8) in November 2022 (Figure 2). Isolation of ETEC began in the pre-monsoon month of May (14.8%), peaked during the monsoon in the month of June (44.3%), and subsequently declined in the following months, with no ETEC isolated in November. Increased levels of ST-ETEC and lower levels of LT-ETEC were observed throughout the study period. The highest combination of LT + ST was detected during the peak summer month of June. Concurrently, as per the Meteorological Department of Bhubaneswar, Odisha, the highest environmental temperatures recorded were 38° C, 39° C, 36° C, and 37° C in May, June, July, and August 2022, respectively. The temperature gradually declined in the subsequent months. The amplification of the *est* gene was higher in June (*n* = 13) and July (*n* = 10) than in May (*n* = 1) and August (*n* = 1).

### 3.7 Antimicrobial resistance in ETEC

**Figure 3:**
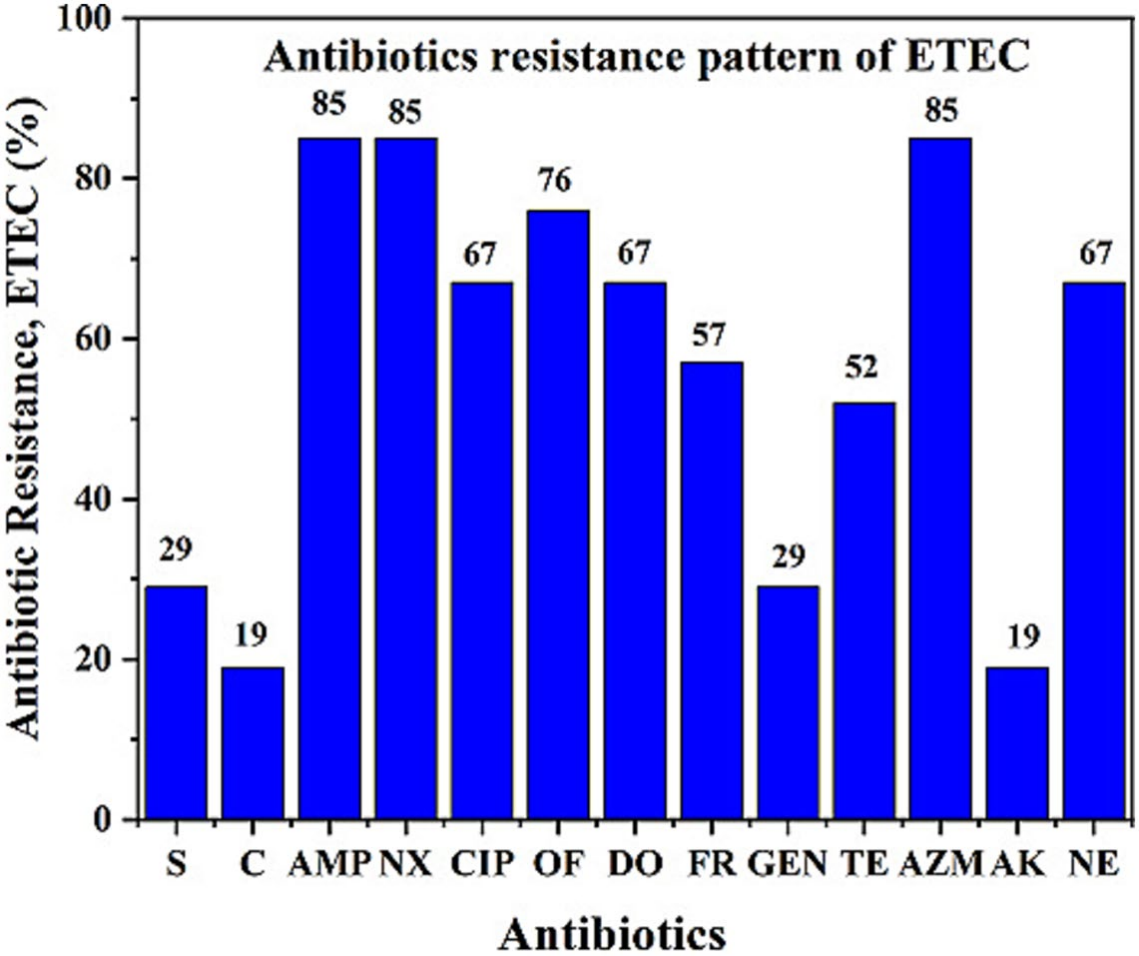
Antibiotic resistance pattern of ETEC isolates showing resistance to different antibiotics. ETEC isolates showed high resistance to several commonly used antibiotics, indicating a concerning trend of multidrug resistance during the study period from May to November 2022.

The antibiotic resistance pattern is shown in Figure 3. The majority of the ETEC strains (95%) were multidrug-resistant (MDR) (resistant to three or more groups of antibiotics). The ETEC antibiotic resistance profile was as follows: tetracycline (TE, 52%), ampicillin (AMP, 85%), neomycin (N, 67%), furazolidone (FR, 57%), norfloxacin (Nx, 85%), ciprofloxacin (CIP, 67%), ofloxacin (OF, 76%), azithromycin (AZM, 85%), and doxycycline (DO, 67%). More than 50% of the ETEC strains exhibited a combination of multiple drug resistance profiles, including AMP, NX, OF, DO, FR, TE, AZM, CIP, and NE. In contrast, the strains were susceptible to S, C, GEN, and AK.

## 4 Discussion

The findings of this study emphasize the significant role of ETEC in the age-specific burden of diarrheal disease. The high prevalence of ETEC among children aged 0–23 months reinforces its established status as a leading cause of diarrhea in infants. This observation aligns with previous research, indicating that infants are highly susceptible to toxigenic *E. coli* due to their immature immune system and frequent exposure to contaminated food and water. Interestingly, the lower prevalence of enteropathogenic *E. coli* (EPEC) and enteroaggregative *E. coli* (EAEC) in younger age groups indicates that these strains may be more commonly acquired in older children and adults, potentially due to differences in exposure pathways and the development of acquired immunity.

### 4.1 Age-specific burden of ETEC

ETEC is a well-established leading cause of diarrhea in infants, particularly affecting children aged 0–23 months. However, despite this peak in infancy, the weak negative overall correlation (*R* = −0.013) indicated that the prevalence of ETEC did not consistently increase or decrease with age across the entire sample. While the present data indicate a relatively uniform distribution, it is critical to consider additional risk factors, such as hygiene practices, dietary habits, and environmental exposure, that may influence ETEC transmission beyond age alone. Furthermore, the limited statistical significance observed in mixed infections could be attributed to sample size constraints, highlighting the need for large-scale studies to gain deeper insights.

Notably, this study reports ETEC as the predominant enteric pathogen causing diarrhea in 2022, replacing *V. cholerae*, which has historically been the leading cause of diarrhea in this region. Similar trends have been observed in other cholera-endemic regions, where fluctuations in enteric pathogen prevalence occur due to environmental and epidemiological factors. The present findings suggest that both children under 5 years of age and adults aged 20–60 years are at the highest risk for ETEC-associated diarrhea, which is consistent with previous studies (Chakraborty et al., 2024; Chandra et al., 2012). Global studies have also highlighted significant geographical variation in ETEC prevalence, with reported rates ranging from 18 to 38% among symptomatic children in countries such as Bangladesh, Mexico, Peru, Egypt, Argentina, Nicaragua, and Tunisia (Rivera et al., 2010; Hien et al., 2008; Bueris et al., 2007). The present study reports an overall ETEC prevalence of 20.3%, with distinct age-group distributions that are consistent with findings from Turkey, Bangladesh, and Zambia (Ozerol et al., 2005; Chakraborty et al., 2024; Silwamba et al., 2022). The severity of ETEC diarrhea was greater in adults, with more cases of dehydration and a higher need for intravenous (IV) rehydration therapy, emphasizing the significant clinical burden of ETEC in the region.

### 4.2 Seasonality of ETEC

The seasonal dynamics of ETEC infections observed in this study further support the established understanding that ETEC prevalence exhibits a seasonal peak. Similar to patterns documented in Bangladesh and Egypt (Hosangadi et al., 2019; Mansour et al., 2014), our data indicate that ETEC incidence peaks in the pre-monsoon season, reaching its highest incidence in June before declining during the rainy months. In addition, variations in toxin phenotype expression were observed, with ST-producing ETEC being more prevalent in children and LT-producing ETEC being more prevalent in adults. This finding is clinically significant, as LT-ETEC infections are associated with more severe dehydration and increased hospitalization rates, resembling cholera-like diarrheal illness in North India (Taneza et al., 2004).

### 4.3 Antimicrobial resistance

Another critical finding of this study is the high prevalence of multidrug-resistant (MDR) ETEC, which raises substantial public health concerns. The study reports that ETEC isolates showed poor response to multiple commonly used antibiotics, including ampicillin, norfloxacin, ofloxacin, doxycycline, furazolidone, tetracycline, azithromycin, and ciprofloxacin. In contrast, they remained susceptible to neomycin, streptomycin, chloramphenicol, amikacin, and gentamicin. The observed 95% treatment failure rate with several first-line drugs underscores the growing challenge of antimicrobial resistance (AMR) and highlights the need for ongoing monitoring. Comparisons with previous studies indicate that MDR ETEC is a rising global issue, with therapeutic efficacy patterns shifting over time (Johura et al., 2024; Huchin et al., 2018). Notably, the reduced effectiveness of fluoroquinolones—commonly reported in India, Bangladesh, and Mexico—is particularly alarming in a tourism-intensive region such as Puri, where travelers may face elevated risk (Huchin et al., 2018; Chakraborty et al., 2001). In addition to ETEC, previous reports from India and neighboring countries have also documented drug resistance trends in other enteric pathogens, such as *V. cholerae*, *Shigella*, and *Salmonella*. The study emphasizes the urgent need to revise local diarrhea treatment protocols in light of the diminished efficacy of fluoroquinolones and macrolides.

The unexpected decline in *V. cholerae* isolation in 2022, coupled with the increased dominance of ETEC, raises important epidemiological questions. Several hypotheses may explain this shift: (a) Improved sanitation and safe drinking water initiatives may have reduced *V. cholerae* transmission while still allowing ETEC to spread via direct contact or contaminated food; (b) climatic factors, such as increasing temperatures, may have influenced bacterial survival and proliferation, favoring ETEC over *V. cholerae*; (c) high historical cholera exposure may have led to increased immunity in the local population, potentially reducing *V. cholerae* incidence while allowing other enteric pathogens to emerge; (d) microbial competition within the gut may be favoring ETEC under current ecological conditions; and (e) oral cholera vaccine (OCV) campaigns may have provided indirect protection against *V. cholerae*, inadvertently enabling ETEC to become the predominant pathogen (Samal et al., 2008; Hosangadi et al., 2019).

## 5 Conclusion

In conclusion, this study highlights a significant epidemiological shift in diarrheal disease patterns in Odisha, with ETEC surpassing *V. cholerae* as the leading cause of diarrhea. The high prevalence of ST-ETEC in children and LT-ETEC in adults, coupled with alarming MDR trends, underscores the urgent need for enhanced surveillance, revised treatment guidelines, and targeted public health interventions. From a vaccine development standpoint, the study’s in-depth analysis of ETEC virulence factors (such as the differential distribution of heat-labile and heat-stable toxins) and its age-specific prevalence offer a roadmap for next-generation vaccine design. Effective vaccine candidates must target the most prevalent toxin types and consider age-related immune responses. The development and deployment of effective vaccines should be prioritized. Improved sanitation, access to clean drinking water, and proper waste disposal are essential for reducing transmission, particularly in high-risk rural and tourist-heavy areas. Future studies should explore the underlying ecological and immunological factors contributing to these observed changes to ensure the implementation of appropriate preventive and therapeutic strategies. This first-time report of ETEC dominance in a historically cholera-endemic region underscores the need for ongoing monitoring to understand its long-term epidemiological impact.

## 6 Study limitations

This study was conducted during the post-COVID-19 period, which posed challenges such as a smaller sample size that might have limited statistical significance. The use of non-random sampling, due to weekly sample collection and logistical constraints, might have introduced selection bias in patient enrolment. Furthermore, the study focused on detecting ETEC enterotoxins (LT, ST, and LT/ST) but did not assess other virulence factors or co-infections with viral and parasitic pathogens, which might have influenced the observed disease patterns. Future studies with larger sample sizes and comprehensive pathogen profiling are needed to validate these findings and provide a more complete epidemiological picture.

## Data Availability

The original contributions presented in the study are included in the article, further inquiries can be requested to the corresponding author.
